# A review of the impact of structural racism on lived experiences of adolescents of African descent: Implications for development, brain structure, and health

**DOI:** 10.1038/s41386-025-02239-4

**Published:** 2025-09-26

**Authors:** Carolina Gonçalves, Ka I. Ip, Cierra A. Stanton, Patricia Bamwine, David R. Williams, Uma Rao, Velma McBride Murry

**Affiliations:** 1 Vanderbilt University School of Medicine, Department of Health Policy, Nashville, TN USA; 2 Vanderbilt University, Peabody College, Department of Human and Organizational Development, Nashville, TN USA; 3 Middle Tennessee State University, College of Behavioral and Health Sciences, Department of Social Work, Nashville, TN USA; 4 Harvard University, School of Public Health, Boston, MA USA; 5 University of California, Irvine, School of Medicine, Department of Psychiatry and Human Behavior, Irvine, CA, USA Children’s Hospital of Orange County, Orange, CA USA

**Keywords:** Developmental biology, Psychology

## Abstract

Structural racism is embedded in nearly every sector of society, creating social and environmental contextual drivers that affect the mental and physical health of minoritized populations. Increasing suicide rates and patterns of early onset of chronic disease have prompted inquiries about the potential effects of structural racism on the overall health and well-being of Black youth. To address this concern, we posed the following questions: (1) In what ways does structural racism filter into and affect the development of adolescents of African descent? (2) Have investigations examined variability in the effects and consequences of structural racism on lived experience and development of Black youth? (3) How does structural racism get inside the skull to affect development including mental and physical health outcomes? and (4) Are there pathways through which family-centered preventive interventions can shape youths’ neurodevelopment to avert the negative consequences of structural racism on their health trajectories? Considerations for future research and clinical practices are offered, with implications to refine the complex and entrenched linkages between structural racism and health disparities among minoritized youth, their families, and communities.

## Introduction

Structural racism, a term rooted in the work of Carmichael and Hamilton [1], describes how societal institutions embed racial inequities through laws, policies, and routine practices, producing differential access to resources and opportunities [1–4]. Braveman et al. [5] describes structural racism as, “so embedded in systems that it often is assumed to reflect the natural, inevitable order of things” [[5], p. 173]. We use the term structural racism to capture how these systemic forces shape disparities in education, housing, healthcare, and beyond. African American youth (descendants of enslaved Black Americans in the United States) and Black immigrant youth (foreign-born or youth with at least one parent or grandparent from African, Caribbean, or Latin American countries) share vulnerabilities linked to structural racism, but their experiences differ due to distinct cultural frameworks and histories [6]. Thus, in this review, we use the term *Black* as a general descriptor for individuals of African descent, while noting distinctions between groups when relevant. This approach also acknowledges both groups’ shared experiences with structural racism, while also recognizing the unique cultural, historical, and immigration-related contexts that may shape developmental and health outcomes. In addition, throughout this manuscript, the terms ‘parents’ and ‘caregivers’ are used interchangeably to encompass the diverse individuals who provide primary care and guidance to children and adolescents, recognizing the variability in familial and caregiving structures.

Structural racism is rooted in historical injustices, such as enslavement, segregation, imperialism, and colonialism, and continues to shape inequities and widen disparities in education, housing, healthcare, criminal system experiences, and beyond. Black youth in the U.S.—whether native-born or foreign-born—share vulnerabilities linked to systemic racism but also differ in cultural backgrounds and experiences. Structural racism is a pervasive determinant of health that adversely affects behavioral, mental, and physical health across the lifespan. For Black youth, these effects manifest in elevated rates of depressive and anxiety disorders, post-traumatic stress disorder (PTSD), substance use, suicidality, as well as physical health conditions such as early age onset of hypertension, obesity, and cardiovascular disease [7]. These behavioral, mental, and physical health outcomes share common neurobiological and stress-response pathways. Specifically, repeated exposure to racism-related stress can dysregulate the hypothalamic-pituitary adrenal (HPA) axis, alter inflammatory processes, and disrupt neural circuits related to self-regulation, emotion regulation, impulse control, and reward sensitivity [8]. These biological disruptions contribute to not only compromised socio-emotional behavioral development but also poor psychological functioning and chronic diseases. A greater understanding of how this phenomenon has been examined in extant studies served as the impetus for this review. We summarize relevant studies that address four guiding questions: (1) How does structural racism influence development and health in youth of African descent? (2) Are effects similar or distinct for Black native-born versus Black foreign-born youth? (3) Through what neurobiological mechanisms does racism “get inside the skull”? (4) In what ways do strength-based cultural asset family-centered preventive interventions mitigate the potential harm of structural racism on Black youths’ health and wellbeing? This review synthesizes studies of Black youth aged 9–17, framing structural racism as a socio-political driver and determinant of neurodevelopment and health outcomes, and explores the potential of cultural-strength-based, family-centered interventions to protect youth from succumbing to the consequences of racism-related stress through the enhancement of resilience.

We begin with an overview of relevant theoretical frameworks and conceptual models that explain how structural racism affects health outcomes, followed by synthesization of extant studies focusing on structural racism and its consequences on health and wellbeing of African American and Black immigrant youth. We present a case study that tests the protective nature of families to enhance adolescents’ decision-making and resilient coping, through the Pathways for African Americans Success (PAAS) programmatic effects on brain functionality. Concluding statements and recommendations for future research and practical applications are offered.

## Overview of Relevant Frameworks and Models

Several theoretical frameworks were selected to guide our review. Bonilla-Silva’s concept of structural racism as a systemic, institutional force underlies frameworks explaining racial disparities in health [9]. Critical Race Theory (CRT) emphasizes the role of race as a social construct that reinforces White dominance and has been applied in research linking racism to adolescent mental health and structural disadvantages. Intersectionality, a central tenet of CRT, explains how overlapping identities—such as race, gender, and immigration status—amplify exposure to racism through multiple and complex systems of subordination operating simultaneously across multiple identities [10]. A CRT–intersectionality explanation of how structural racism impacts the mental and physical health of minoritized youth might focus on the consequences of overlapping systems of oppression based on prescribed social positions—for example, suicide risk among Black youth residing in U.S. regions with historical legacies of Jim Crow laws [11].

**Fig. 1:**
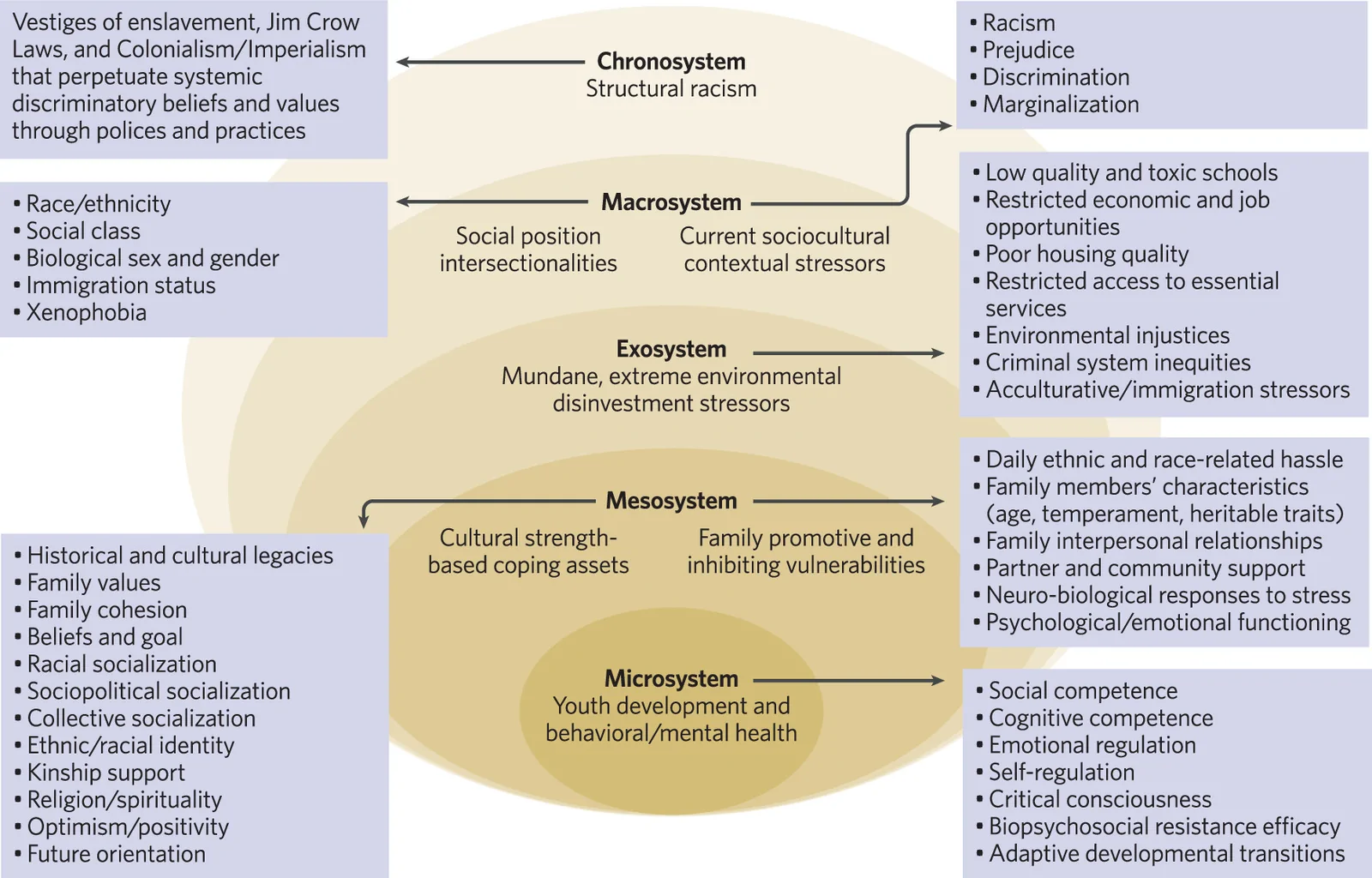
Key indicators of the Biopsychosocial Integrative Systems Model of Cascading Effects of Structural Racism on Black Youth.

Bronfenbrenner’s bioecological model describes how macrosystemic forces cascade through social contexts, shaping microlevel processes that affect youth development [12]. In addition, the *R3ISE Integrative Model* (Racism + Resilience + Resistance Integrative Study of Childhood Ecosystem) and the *Integrative Model for the Study of Stress in Black Families (IMS2BF)* are informative as they both highlight how intergenerational trauma, resilience, and cultural coping assets influence race-related stress responses. However, these models have not been applied in research to explain the ways in which structural racism shapes Black immigrant youth development. To expand the scope of this line of research, we propose a *Biopsychosocial Integrative Systems Model* (BISM) to capture the historical origins and contemporary consequences of structural racism on Black youth, emphasizing intersectionality (e.g., race, ethnicity, and immigration status) as key processes shaping developmental and health trajectories [13] (see Fig. 1).

In sum, despite advances in theoretical frameworks that seek to explain how and why structural racism affects the development, behavioral, mental, and physical health of native born Black American youth of enslaved descent, the experiences of Black immigrant youth remain undertheorized [13, 14]. Applying the model illustrated in Fig. 1 to Black immigrant youth, we view structural racism in the U.S. as historically experienced by descents of enslaved Black Americans and also in parallel with colonialism, imperialism, and U.S.---led military interventions in their home countries [13]. In the following section, we use aspects of selected theories and models to frame our review and synthesis of existing studies.

## The Cascading Effects and Consequences of Structural Racism on The Development and Adjustment of Black Youth

The potential consequence of structural racism begins early in the lives of all Black youth and continues across the life course [15]. Black youth face systemic disadvantages through policies that perpetuate inequality in housing, education, healthcare access, and other racialized disparities. All developmental domains are influenced by structural systems and practices that shape the everyday lived experiences of Black youth, their families, and communities [12]. These circumstances create multiple disparities that hinder opportunities for optimal development. Intersectional factors—namely race, gender, ethnicity, immigration status, geographic residence, and social class—are core rather than peripheral contextual influences on the developmental and health trajectories of Black youth [16]. Eco-socio-political-environmental contexts and processes also play key roles in adolescents’ development [17]. For example, neighborhoods, schools, social media, and the healthcare system are settings where youth are more likely to be exposed to the harms of structural racism and elevated risk for depression, anxiety, and suicide [18, 19]. Studies examining how neighborhoods and built environments filter into the lives of youth of African descent are discussed in the following section.

## Neighborhoods and Built Environments

Neighborhoods and built environments—defined as the physical, social, economic, and environmental conditions where people live, work, and play—affect health and well-being through housing quality, access to essential resources, and socio-environmental safety [18, 20]. Theoretical foundations from CRT and bioecological systems theory highlight that neighborhoods and built environments reflect residents’ social positions, with cascading developmental consequences [10, 12].

Many youths grow up in communities with concentrated poverty due to structurally and systematically planned residential exclusion policies and practice, historically referred to as redlining. Redlining was a banking practice to restrict mortgages granted to Black Americans, controlling areas where they were able to live and purchase homes [20]. Although redlining was outlawed in 1968, its harmful legacy persists, as families continue to face challenges raising children in communities with starkly fewer amenities, even 50 years later [20, 21]. Segregated Black communities of concentrated poverty confront restricted access to resources, food insecurity, healthcare and pharmacy deserts, poor housing quality, failing schools, physical and environmental blight, and high levels of crime and violence. These conditions lead to poor health outcomes and fragile future opportunities [22–27].

Poor housing quality has far-reaching health consequences including increased likelihood of heavy metal (lead) poisoning and vulnerabilities to asthma, cognitive, and neurological problems [28–30]. A systematic review highlighted the high prevalence of lead exposure among African American children, children from low-income households, and those growing up in rural communities, with dramatic increases among these groups over the last few decades [31]. In addition, limited green space access is associated with elevated risk for neuropsychiatric disorders such as developmental disabilities, learning impairments, intellectual disabilities, attention-deficit/hyperactivity disorder (ADHD), and autism [32, 33].

The intersection of segregated race/ethnicity, housing, and poverty also increases risk for violence exposure, firearm injuries, and homicide among Black youth, ages 15–24. Thus, where one lives increases the likelihood of being disproportionately affected by toxic-built environments and environmental injustice, elevating risk for negative mental, behavioral, and physical health outcomes [29, 31, 34].

Research examining neighborhood environment and housing patterns among Black immigrants is sparse. Available studies suggest that immigrants from Caribbean, Latin America, Ethiopia, Eritrea, and Somalia often settle in neighborhoods experiencing an influx of White residents and a decline in African American populations [35]. These settlement patterns have been described as a “social buffer” that shifts racial tension between African American and White residents [35]. However, such shifts reflect the enduring legacy of anti-Black racism more than genuine integration. Rather than addressing discriminatory housing policies, displacement patterns may further marginalize both immigrant and native-born Black communities, by limiting their long-term stability and access to resources, ultimately harming adolescents’ mental and physical health. While these findings offer insights on the cascading effects of structural racism on Black youth, more research is needed to examine to disentangle the experiences of Black immigrant youth growing up in segregated neighborhoods, including immigrant enclaves and predominantly White or Black neighborhoods. Black immigrant youth living in either of these neighborhoods may have different developmental and health outcomes in comparison to native-born Black youth.

While redlining is often associated with urban dwelling, recent changes in rural spaces driven by economic transformations and environmental shifts have created conditions reflective of what some have characterized as “rural ghettos” [36]. Moreover, the establishment of AI research and data centers in rural communities often exemplifies environmental injustice, particularly when these facilities are situated in historically Black neighborhoods with a legacy of environmental degradation [37]. The emergence of AI facilities in rural communities, powered by unpermitted gas turbines, has been linked to increased respiratory issues among residents, including a 79% rise in nitrogen dioxide levels [37].

Such environmental exposures are particularly detrimental to children and adolescents, whose neurodevelopment and mental health are highly sensitive to pollutants. Research indicates that air pollution can impair brain development, leading to cognitive deficits and behavioral problems [38]. Additionally, systemic factors like socioeconomic status and inadequate regulation exacerbate the risks for marginalized communities, increasing their vulnerability to environmental hazards. Just as exposure to environmental hazards can compromise youths’ neurodevelopment and both mental and physical health, the harmful consequences of resegregation for Black youth are also evident in schools, the criminal system, hospital settings, social media, and beyond.

## Structural racism consequences of resegregation

### School

Many Black children attend under-resourced schools with poor air quality, aging infrastructure, overcrowded classrooms, limited access to mental health services, and are exposed to racialized oppressive experiences in these spaces. Research on the school experiences of Black youth have shown that they are more likely than White peers to attend schools with fewer opportunities. They are also more likely to be excluded in advanced placement classes and instead tracked into special education, often without formal assessment. These inequities contribute to academic failure and to the school-to-prison pipeline disparities [39–41].

Although the United States has become more diverse, patterns of increased school resegregation have intensified [42], despite the celebrated success of Brown vs. Board of Education [43]. Resegregation has been linked to mistreatment of Black children and adolescents in schools. Although schools should be safe spaces, they are often environments where racialized and oppressive environmental experiences begin as early as kindergarten [19, 40, 44–46].

Paulle describes racialized school environments as “toxic schools,” where youth are exposed to threatening, violent, and hazardous conditions that undermine academic, mental, and physical health [19]. Within these schools, Black students often face discriminatory policies, including excessive detention, suspensions, and expulsions [19, 39, 47], are common occurrences in toxic schools. With 87% of elementary and secondary teachers identifying as White, the likelihood of racism exposure is high [48, 49]. These toxic environments take a toll on Black youth, leading to compromised mental health functioning including anxiety and depressive symptoms, along with behavioral problems, social isolation, disengagement, disconnection, academic failure, and early school dropout [50–55].

Comparatively, there are distinct educational challenges experienced by foreign born versus native born Black youth. Foreign-born, or Black immigrant youth may be labeled as “good students” under the stereotype of hardworking immigrants or as “bad students” when associated with stereotypes of Black native-born youth [56]. This binary categorization, rooted in structural racism, perpetuates anti-Black ideologies that harm all Black youth, regardless of ethnicity or origin. Black immigrant students often face dual racial positioning–-cast as a “model minority,” while simultaneously assumed to have low academic proficiency. This tension can create conflict with their native-born peers. Like their Black native-born counterparts, Black foreign-born students are subjected to low expectations, limited opportunities, and cultural marginalization by White teachers. They also experience othering from Black native-born peers based on language or country of origin [56–59]. This dual marginalization can foster maladaptive coping strategies. For example, discriminatory treatment from teachers may lead to truancy, isolation, low school belonging, and alienation, with increased risk for depression and anxiety, with negative spillover effects on school performance [60–63].

Overall, research highlights how schools, both as built and social environments, filter structural racism into the lives of Black youth. Broader systemic policies and practices create ideologies that shape interactions with teachers, staff, and peers. These experiences can affect multiple domains of development, including mental, emotional, cognitive, and physical health, with lifelong consequences [54]. These experiences have also been associated with increased vulnerabilities that include contributing to the school-to-prison pipeline [64].

### Criminal system

The U.S. criminal system perpetuates structural racism through policies such as ‘zero-tolerance,’ giving rise to harsh and racially biased disciplinary practices in schools that disproportionately affect Black youth [65]. As a result, Black youth are disproportionately incarcerated in juvenile systems and experience higher rates of arrests and detentions for the same offenses as their White counterparts [66]. The criminalization of school discipline —reporting students for minor misbehavior that could be managed by school administrators— is a major entry point into the juvenile system [66]. These disciplinary practices occur not only in schools but in Black neighborhoods [67, 68]. Heighten law enforcement surveillance, including racial profiling directed at Black youth, perpetuates discriminatory practices that have been linked to the mass incarceration of Black youth [65].

Evidence indicates that interactions with law enforcement constitute adverse childhood experiences for Black youth, with gendered differences such that Black boys report more frequent, hostile, and anxiety-inducing encounters compared to Black girls [69]. Even vicarious exposure—such as viewing images of police violence against Black people—elicits heightened sympathetic nervous system (SNS) responses, emotional distress, and long-term health effects, including asthma and early onset of cardiovascular disease [70, 71].

Developmentally, Black youths’ involvement with the criminal justice system often begins at a very early age, with some children being arrested and detained as early as age 10. One early pathway into the justice system is through Child Protective Services (CPS). Black families are disproportionately involved with CPS because of structural racism [72, 73]. Thomas et al. (2023) highlight racialized poverty—driven by state and city policies—as a major predictor of CPS exposure. Structural racism, therefore, functions as a social driver of economic deprivation, shaping families’ capacities to provide for their children’s needs and wellbeing.

Low-income families, who are likely to rely on social services, face heightened surveillance and scrutiny, increasing the risk for CPS reports, increasing likelihood of contact with the criminal system [74]. To address this problem, Thomas et al. (2023) call for multi-prong policy changes that target the root causes of racialized economic deprivation. Reforming CPS will require eliminating the criminalization and policing of Black families under the guise of child protection. Equally important is the inclusion of families’ voices in CPS reform efforts.

Haitian families’ experiences illustrate the need for system level changes. Despite concerns about mistreatment of their children in schools by peers, school officials, and law enforcement, Haitian parents identified several barriers that prevented them from protecting their children [59]. Fear of retaliation due to immigrant status and cultural norms that emphasize obedience to authority limited their ability to navigate CPS and the juvenile justice system. These dynamics, while reported by one ethnic group, have implications for other Black families, given the pervasiveness of structural racism in U.S. systems. These experiences impair parents’ ability to support their children, increasing risks for youth depression [75–77], disrupted identity development, and emotional dysregulation. They also contribute to accelerated vulnerability to early-onset chronic disease [78–80] and are further reinforced through social media.

### Social media

Social media represents another environment where Black youth encounter racial discrimination and oppression [81–83]. Algorithms shape these digital spaces, often reinforcing stereotypes and marginalization [84, 85]. Drawing on the theoretical underpinnings of frameworks guiding our review, the work of Daniels is informative to explain how racism is perpetuated in social spaces. Daniels describes social media as “spaces where race and racism play out in interesting, sometimes disturbing ways, reshaping racist dynamics through affordances, policies, and overt discrimination” [[86], p. 70].

Quintana and McKown highlight the effects of vicarious racial discrimination, while Maxie-Moreman and Tynes found that seeing people of one’s own racial group harassed, attacked or harmed online is associated with increased risk for depression, anxiety, post-traumatic stress disorder, and substance use disorder among Black youth [83, 87, 88]. Chronic exposure to such content repeatedly activates stress responses, which over time leads to “weathering” of both body and mind [89, 90]. On the other hand, social media can provide positive experiences, particularly supporting ethnic–racial identity development among native-born Black youth. One study, for example, found that Black adolescents with stronger online connections to same-ethnic peers reported more positive ethnic–racial identity [91].

In fact, research on Black foreign-born youth has primarily emphasized social media benefits, such as connecting with cultural heritage [13]. However, little is known about the social media harms they face. Given their exposure to similar stereotypes as their Black native-born peers, additional research is urgently needed to examine how online racism affects their development and mental health, and how interventions to promote digital interventions for well-being can be integrated into health and clinical care services for easy access. It is noteworthy, however, that accessing services in healthcare systems may be met with exposure to race-related incidents [92].

### Health care

From the 17th century onward, enslaved Black people were denied proper medical care and instead developed a “slave health subsystem,” which was underfunded and used only in emergencies [93]. Throughout history, Black individuals have been exploited in medical research without consent, most infamously in the Tuskegee Syphilis Study [94–96]. These violations fostered mistrust between Black communities and health professionals.

Health care access and quality for Black populations continue to lag behind that of White populations, with worse outcomes across major diseases [97–99]. Policies such as the Hill-Burton Act of 1946 reinforced segregation in health care for decades [100], worsening disparities. Today, Black immigrants initially report better health outcomes than U.S.-born Black individuals [101, 102], but their health declines with increased length of residence in the U.S. [103]. This suggests that growing up in a racialized society elevates health risks for Black youth residing in the U.S., both native-born and foreign-born.

Mental health care exemplifies some of the most pronounced consequences of structural racism, producing significant inequities. Black youth are less likely than White youth to receive necessary mental health services, despite having comparable or greater levels of need [39–41]. When services are accessed, they are often of lower quality, with fewer evidence-based treatments offered and greater rates of early termination [104, 105]. Structural barriers—including cost, lack of insurance coverage, transportation, and shortage of culturally competent providers—compound these disparities [100]. Moreover, racial bias in the health care system contributes to systematic underdiagnosis of mood disorders and overdiagnosis of behavioral disorders among Black youth [36, 106–108], leading to punitive rather than therapeutic interventions. For Black immigrant youth, stigma surrounding mental health and lack of culturally tailored services further reduce access and engagement [101–103].

Promising approaches are beginning to address these gaps. Community-based interventions such *The Strong African American Families*, *The Pathways for African Americans Success*, and *Healing Ethno-Racial Trauma, Culturally Informed Parent Training* programs that partner with trusted institutions—such as schools, Black churches, and grassroots organizations—have shown success in increasing access and engagement in behavioral and mental health care [76, 109, 110]. Culturally adapted therapies such as Cognitive Behavioral Therapy (CBT) modified to incorporate racial socialization, identity development, and experiences of discrimination, also have demonstrated effectiveness in promoting wellbeing and improving mental health outcomes for Black youth [76, 109, 110]. In addition, school-based mental health programs that employ diverse providers and embed services directly in educational settings reduce barriers related to stigma and access [111]. For Black immigrant families, interventions that incorporate cultural values, family narratives, and language-specific resources have improved both retention and mental health treatment efficacy [112]. Together, these models illustrate the importance of designing and scaling mental health services that are responsive to cultural context and structural inequities that affect the wellbeing of Black youth. They also demonstrate effective ways to circumvent negative experiences and treatment of Black youth within the health system.

Their experiences in health systems have been described as a “core determinant of child health” [6, 113]. Race-related experiences with physicians, nurses, and staff discourage help-seeking and engagement with care, contribute to misdiagnoses, and perpetuate inequities [36, 106–108]. Addressing these disparities requires pediatricians, clinicians, and behavioral and physical health professionals to not only document the cumulative effects of systemic racism on their patients’ health but to also advocate for structural change.

## How Does Structural Racism “Gets Under the Skull”

Structural racism not only sustains inequities but has been increasingly recognized as a key determinant of health and a socio-political driver of racial-ethnic disparities [114]. While its social and environmental consequences are well- documented, the biological mechanisms through which structural racism affects health remain understudied, especially among Black immigrant youth. The following sections will examine existing evidence on how structural racism shapes neurodevelopment, particularly in African American children and adolescents. We highlight several interrelated but underexplored biological pathways through which structural racism may influence neurodevelopment and health outcomes, including accelerated brain aging, dysregulation of the HPA axis, allostatic load, neuroinflammation, and epigenetic age acceleration. These mechanisms provide a framework for understanding how systemic inequities become biologically embedded, contributing to long-term physical and mental health disparities. By clarifying these pathways, this review seeks to inform the development of targeted, mechanism-driven preventive interventions to mitigate mental health inequities.

Research on neurocognitive and mental health disparities often attributes differences to race or family-level adversity, such as socioeconomic status or adverse childhood experiences. However, these explanations overlook systemic forces like structural racism—manifested through factors such as residential segregation, inequitable policies, and intergenerational wealth disparities—that fundamentally shape brain development and mental health. Emerging evidence highlights how neighborhood environments, shaped by structural racism, influence neurodevelopment. Access to well-funded schools, safe environments, and stable housing supports healthy brain development, while structural racism systematically restricts these resources for racially minoritized communities. Simultaneously, these communities face disproportionate exposure to harmful environmental conditions like air pollution and lead toxicity. The consequences are profound: children aged 9–10 years in very low-opportunity neighborhoods face a 40% higher risk of death and are 60% more likely to experience caregiver loss, both of which have lasting effects on brain development and mental health [115].

Living in underserved neighborhoods significantly impacts brain development. A systematic review [116] identified 37 studies linking neighborhood conditions to brain structure, consistently finding that lower socioeconomic conditions are associated with smaller brain volumes [117, 118], thinner cortices [118–121], and reduced white matter integrity [122]. Crucially, racial disparities in brain structure are largely explained by neighborhood disadvantage rather than race itself. For example, neighborhood deprivation mediates differences in brain volume and cortical thickness between Black and White youth [79, 123–125]. These structural changes also link neighborhood disadvantages to mental health outcomes, such as externalizing symptoms and cognitive impairments [126, 127].

To understand how structural racism becomes biologically embedded, Muscatell et al. [29] proposed a model linking racism to neural and physiological mechanisms, highlighting how racism activates the sympathetic nervous system (SNS) and the HPA axis, leading to increased neuroinflammation and negative health outcomes [29]. Chronic activation of these systems places individuals, particularly African Americans, in a heightened state of “fight or flight,” a coping mechanism that contributes to long-term biological wear and tear. While this model provides a valuable foundation, it primarily focuses on adults, leaving a critical gap in understanding how these processes operate during neurodevelopment in children and adolescents. Building on this framework, the following sections explore specific pathways through which structural racism could affect neurodevelopment in children and adolescents, including accelerated brain aging, dysregulation of the HPA axis, allostatic load, neuroinflammation, and epigenetic modifications. Each pathway highlights how systemic inequities manifest in the brain and body, with profound consequences for health and well-being.

### Accelerated brain aging

Exposure to chronic stress and adversity, particularly in the context of structural racism, can lead to accelerated biological aging, a process characterized by premature deterioration of cellular and physiological functions. The stress acceleration hypothesis posits that in response to unpredictable and threatening environments, the brain adapts by expediting neurodevelopment [128, 129]. This adaptation may confer short-term survival benefits but at the cost of long-term neural plasticity and resilience. Youth growing up in neighborhoods characterized by socioeconomic deprivation and violence often exhibit earlier reductions in cortical thickness and gray matter volume in regions critical for cognitive and emotional regulation, such as the prefrontal cortex and hippocampus [117, 130]. These neuroanatomical changes have been linked to poorer executive function and greater difficulty in regulating emotions [131]. These changes may reflect an adaptive response to high-threat and/or unpredictable environments, enabling faster emotional and behavioral responses to danger. At a functional level, early adversity may drive premature maturation of the corticolimbic circuitry, which is involved in stress responses and emotion regulation. For example, youth exposed to chronic stress exhibit more adult-like connectivity patterns between the amygdala and prefrontal cortex [132–134], which may reduce anxiety in the short term but limit long-term emotional flexibility [132, 135]. In addition, a more mature corticolimbic connectivity pattern may buffer against the negative effects of neighborhood deprivation on internalizing symptoms among adolescents over one year [136]. While these neurodevelopmental adaptations may enhance immediate survival in environments characterized by high threat and unpredictability, they may also compromise long-term cognitive and emotional functioning, potentially increasing vulnerability to mental health challenges such as anxiety and depression later in life [128, 137, 138]. However, direct evidence linking accelerated brain maturation to long-term psychological or cognitive outcomes remains limited. Most existing research is cross-sectional or short-term in nature, and there is a notable lack of longitudinal studies—particularly those that include racially and ethnically diverse populations such as African American or Black immigrant youth. Future longitudinal research is critically needed to examine whether and how these early neurodevelopmental shifts shape cognitive and emotional trajectories across adolescence and into adulthood, particularly in populations disproportionately exposed to structural adversity.

### Dysregulation of the HPA axis

The hypothalamic-pituitary-adrenal (HPA) axis, a central regulator of the body’s stress response [139], plays a critical role in mediating the effects of chronic stress on neurodevelopment. Chronic stress, including that arising from structural racism, immigration- related stressors, and adverse childhood experiences, can lead to prolonged HPA axis activation. This results in persistently elevated cortisol levels or, conversely, blunted cortisol responses, both of which are maladaptive [140]. Cortisol, being lipophilic, can cross the blood-brain barrier and directly influence brain structure and function by binding to glucocorticoid receptors (GRs) and mineralocorticoid receptors (MRs) in stress-sensitive regions such as the prefrontal cortex, hippocampus, and amygdala [141]. Excessive cortisol levels in childhood have been linked to reduced hippocampal volume, which correlates with impairments in memory and learning [142]. Chronic stress also leads to dendritic atrophy and synaptic loss in the prefrontal cortex, impairing executive functions such as decision-making, impulse control, and emotion regulation [143]. Dysregulation of the HPA axis increases vulnerability to mental health disorders such as depression and anxiety. Individuals with a history of childhood maltreatment exhibit heightened amygdala reactivity to threat, blunted ventral striatal response to reward, and weakened connectivity between the prefrontal cortex and amygdala [138]. These neurobiological alterations contribute to long-term cognitive and emotional challenges.

### Allostatic load

The Allostatic Load Model (ALM) provides a framework for understanding how prolonged stress contributes to disease risk by causing cumulative biological strain [144]. Allostasis, the body’s ability to maintain physiological stability through dynamic adjustments in stress- mediating systems, is essential for short-term adaptation. However, chronic exposure to stress necessitates repeated activation of these systems, leading to allostatic overload—a state of physiological wear and tear that increases susceptibility to both mental and physical health disorders [130, 144]. There is growing evidence that allostatic load begins accumulating early in life. Children exposed to maltreatment or chronic adversity exhibit enduring changes in the nervous, endocrine, and immune systems, including reduced volume in the prefrontal cortex and hippocampus, heightened activation of the HPA axis, and elevated levels of inflammation, signaling early biological wear and tear [145]. Longitudinal studies also link cumulative adversity in childhood to higher allostatic load during adolescence [146].

Stressors associated with structural racism, including discrimination, economic hardship, and exposure to neighborhood violence, can lead to progressive wear and tear and accelerate allostatic overload. For instance, Black native-born youth who reported higher levels of perceived discrimination and lower levels of emotional support between ages 16-18 showed significantly higher allostatic load by age 20 [147]. This dysregulation spanned multiple physiological systems, including elevated cortisol levels, higher blood pressure, increased body mass index, and heightened inflammatory markers, even after accounting for socioeconomic factors. These findings underscore how racially patterned stressors during adolescence can become biologically embedded, increasing risk for chronic disease and psychiatric illness in adulthood.

In addition, the skip-deep resilience hypothesis [148, 149] offers a compelling framework for understanding the complex interplay between psychosocial success and physiological vulnerability. This hypothesis posits that Black Americans from disadvantaged backgrounds who display outward markers of resilience—such as better self-control, lower rates of substance use, higher academic achievement, and upward mobility—may nevertheless incur hidden biological costs [149–151]. Despite exhibiting strong psychosocial functioning, longitudinal studies have revealed that these individuals often demonstrate higher levels of allostatic load and biomarkers of accelerated aging [149–151], suggesting that success under chronic stress may come at the expense of physical health.

More research is needed to examine the skip-deep resilience phenomenon, and the broader allostatic load framework extends to Black immigrant youth, who face compounded stressors stemming from both racial and ethnic discrimination as well as xenophobic exclusion. Understanding how these intersecting forms of structural adversity shape both visible resilience and hidden physiological costs is essential for developing more nuanced and equitable models of adaptation and health.

## Neuroinflammation and Immune Dysregulation

Chronic stress, including that induced by structural racism, activates the immune system and triggers the release of proinflammatory cytokines such as interleukin-6 (IL-6) and tumor necrosis factor-alpha (TNF-α). These cytokines influence neural processes through multiple pathways, including vagal nerve signaling and microglial activation, leading to neuroinflammation [152]. Once in the brain, proinflammatory cytokines activate microglia, the brain’s resident immune cells, which then release further proinflammatory molecules. This cascade of neuroinflammation disrupts neural circuits, damages neurons, and alters brain function. Chronic microglial activation is linked to synaptic pruning, reduced neurogenesis, and impaired neural plasticity, all of which contribute to cognitive and emotional dysfunction [153, 154]. Studies indicate that interpersonal discrimination is associated with heightened inflammatory responses in both youth and adults. For example, Black native-born youth who report greater racial discrimination exhibits increasing levels of inflammatory markers over time, with Black male adolescents showing particularly strong proinflammatory responses [155]. Black immigrant youth navigate structural racism alongside immigration-related stressors, both collectively serving as key contributors to chronic stress [112]. Therefore, more research is needed to disaggregate these experiences within and across different groups of youth of color to better identify mechanisms that can promote and improve mental health outcomes, particularly for those that are disproportionately affected by the consequences of race-and-immigrant related stress.

### Epigenetic modifications

Epigenetics refers to environmentally induced changes in gene expression that do not alter the underlying DNA sequence. One of the most studied epigenetic mechanisms is DNA methylation, where the addition of a methyl group to DNA typically suppresses gene expression. Structural racism can accelerate biological aging through epigenetic changes, leading to epigenetic age acceleration—when an individual’s biological age exceeds their chronological age. For example, Black native-born and Hispanic foreign-born youth exposed to systemic stressors, such as adverse school conditions or police intrusion, exhibit greater epigenetic age acceleration than their White peers [156, 157]. This premature biological aging increases risks for chronic diseases, cognitive decline, and mood disorders.

Critically, epigenetic modifications can also be transmitted across generations, providing a biological mechanism for the intergenerational transmission of trauma. Much of the evidence on intergenerational epigenetic effects comes from populations exposed to severe collective trauma, such as Holocaust survivors and their offspring, who show inverse patterns of DNA methylation in the FKBP5 gene, which regulates the body’s stress response [158]. These findings suggest that severe trauma leaves a lasting molecular imprint, shaping stress regulation and mental health across generations. Although no studies to date have directly linked specific epigenetic signatures to the legacy of systemic racism in African American populations, the cumulative impact of enslavement, segregation under Jim Crow laws, systemic violence, and mass incarceration constitutes a profound form of intergenerational trauma, which may influence gene expression through epigenetic changes. This represents a critical area for future investigation.

Black immigrant populations also face unique epigenetic vulnerabilities. Youth from refugee backgrounds may carry the biological imprint of pre-migration trauma—such as war, forced displacement, and extreme poverty. Evidence from other contexts underscores this possibility: epigenetic studies in Rwandan adults suggest that prenatal exposure to genocide-related trauma leaves lasting molecular signatures. Rivera et al. [159] found that conception through genocidal rape was linked to DNA methylation differences in *BDNF* and *SLC6A4*, which are involved in neural plasticity, stress regulation, and mood [159]. Methylation at these sites was also associated with depression and anxiety symptoms. Uwizeye et al. [160] reported accelerated epigenetic aging among similarly exposed individuals, particularly those conceived through rape, with effects amplified by ACEs [160]. Musanabaganwa et al. [161] identified methylation changes in *BCOR*, *PRDM8*, and *VWDE*, with some patterns present in both blood and brain tissue, suggesting potential neurodevelopmental relevance [161]. It is noteworthy, however, that all three studies are cross-sectional, rely on retrospective exposure data, and use single methylation measures, which limits causal interpretation. In sum, more research is needed to elucidate how diverse forms of sociopolitical and historical trauma become biologically embedded and passed on intergenerationally—particularly through longitudinal, multi-generational, and epigenomic studies of both Black native-born and Black foreign-born families.

Collectively, studies identifying pathways through which structural racism gets inside the skull highlight biological embedment as a key mechanism that shapes neurodevelopment and mental health trajectories through multiple interrelated pathways. Importantly, these findings challenge the notion that racial disparities in brain structure and function are biologically inherent. Instead, they highlight how racism, rather than race itself, drives disparities in neurodevelopmental outcomes. Understanding these biological pathways, particularly among populations facing cumulative exposures to structural racism and xenophobia, is essential for developing interventions that mitigate the health impacts of systemic inequities. Policies that expand access to high-quality education, safe housing, social safety net programs, and environmental protections are likely critical to reducing the biological toll of racism and promoting health equity.

Despite the advances in this field, significant gaps remain. Much of the existing research is cross-sectional, limiting the ability to establish causality or disentangle bidirectional relationships between neurodevelopment and health. Future studies should prioritize longitudinal designs to better capture how structural racism dynamically influences biological processes over time. Moreover, given the importance of developmental timing and sensitive periods in shaping biological systems [117], future studies are needed to examine how structural racism may interact with these critical windows—such as infancy, early childhood, and adolescence—to amplify its effects on long-term health outcomes. As Iruka, Shonkoff, and Curenton [162] argue, uncovering how racism and structural inequities shape developmental and biological processes requires a comprehensive, multi-level approach, as proposed in R3ISE, and is also illustrated in our model (Fig. 1). This involves tracing their early roots—often beginning prenatally—and examining how their effects accumulate, compound, and are transmitted across generations throughout the lifespan.

Additionally, research should expand beyond individual-level factors to examine broader systemic interventions that can buffer the impact of structural racism on neurodevelopment. By integrating neuroscience, social epidemiology, and policy research, future work can move beyond documenting disparities to developing evidence-based strategies for reducing them. Moreover, addressing the biological consequences of structural racism requires a multidimensional approach—one that not only seeks to mitigate adversity but also to foster environments that promote resilience, well-being, and equitable opportunities for all children and adolescents. The studies included in the current summary did not distinguish Black foreign-born youth from Black native-born youth. However, thus far, two key themes have emerged:(1) structural racism creates and sustains adversities for Black youth and their families who reside in the US, regardless of country of origin, and (2) many Black youth have the capacity to overcome the consequences of structural racism and its socio-eco-political risks, through the cultural strength based protective nature of Black parents’ caregiving practices [76, 109, 110].

## The Protective Nature of Parenting in Black Families

Demands and challenges associated with built and social environments that perpetuate structural racism policies and practices can compromise caregivers’ psychological functioning and shape how minoritized parents help their children navigate race-related stressors. Parents and other family members serve as the primary sources of socialization and as filters through which messages about the broader culture are affirmed, challenged, and interpreted. Although cultural socialization is universal, minoritized and immigrant youth must be socialized into their ethnic culture, the dominant culture, and learn to resolve inconsistencies and conflicting viewpoints across cultures [163].

Acculturation describes the experiences of individuals who, despite migrating and adopting the language, values, and behaviors of the host culture, remain marked as “different” due to distinct phenotypic characteristics [164]. For Black immigrant families, acculturation may take a tri-dimensional form as individuals navigate their own culture alongside Black and White native-born Americans cultures [165]. As parents employ strategies to support their children, these approaches evolve with experience. For instance, many Black immigrant parents socialize their children to embrace their ethnic identity while distancing themselves from identifying with Black native-born, as a protective strategy to buffer them from racial discriminatory experiences [58]. However, such approaches—often influenced by anti-Blackness or limited understanding of structural racism—may weaken Black foreign-born youths’ racial/ethnic identity. This parenting strategy may leave their children less equipped to navigate the consequences of that they will experience growing up in a society marked by racism discrimination and unintentionally placing them at increased risk for poor mental health outcomes. Research shows that racial identity serves as a critical protective factor for Black native-born youth, fostered through parental racial/ethnic socialization, and is also linked to numerous positive prosocial outcomes, including lowering risk for depression and anxiety [166, 167]. Maintaining a strong orientation to one’s racial-ethnic group and culture of origin is therefore crucial to mitigating the potential negative impact of structural racism on Black youths’ mental health. For Black foreign-born youth, fostering both a strong racial- ethnic and country of origin identity through parenting practices is equally important, and, similar to parenting strategies of Black native-born parents, parenting strategies that equip their children to be aware of and effective coping skills to navigate racial discrimination [13].

Overall, protective caregiving practices in Black families—particularly among those that are native-born— have been identified as salient, malleable targets in strength-based family preventive interventions. For example, Murry et al. (2023) tested The Strong African American Families (SAAF) Program, which aimed to enhance parental socialization strategies to protect children from succumbing to the harm that can occur through racialized experiences in school settings. Results showed that exposure to SAAF enhanced parental academic race-related socialization practices, which was indirectly associated with academic success of their children through the enhancement of racial pride. Further, these protective processes were most pronounced among youth experiencing high levels of racial discrimination in school [109].

Similarly, using the IMSSBF conceptual framework [168], which connects historical and contemporary structural racism to child behavioral health outcomes, Berkel and colleagues (2024) evaluated The Pathways for African Americans Success (PAAS) program [76]. Results demonstrated positive program effects on racial equity-informed parenting and racial pride, buffering adolescents’ mental health against exposure to racial discrimination. These findings underscore the importance of engaging families in treatment and, more critically, of incorporating strength-based, culturally responsive interventions to address racial stress. At the systems level, school-focused interventions can further promote safe, inclusive environments that counteract structural racism through equitable policies [76].

Given these promising findings, a critical question remains for advancing both neurobiological and prevention science research: Which neurobiological mechanisms underlie the PAAS program’s intervention-induced changes in youth protective factors that effectively influence behavioral outcomes and neural circuitry? We explore this question through a case study of the PAAS e-Health program.

### Case study example: Testing PAAS e-Health effects on brain functionality and risk prevention among Black youth

Neuroimaging studies implicate deficits in the fronto-striatal circuit in heightened risk-taking behaviors. However, the role of functional coupling between the reward system (ventral striatum; VS) and regions responsible for emotional and cognitive control (prefrontal cortex; PFC) in adolescent coping remains poorly understood. Even less is known about how exposure to preventive psychoeducational interventions affects VS-PFC connectivity. Understanding these mechanisms may be critical for decision-making, self-regulation, and coping with developmental challenges, including the consequences of structural racism.

The Pathways for African Americans Success (PAAS) program was evaluated in a three-arm randomized trial (technology-based e-Health, traditional small group, and literature control) involving 418 sixth graders and their primary caregivers. Families were randomized to technology (*N* = 141), group (*N* = 141), or literature control (*N* = 136). Families assigned to the PAAS e-Health condition received the program on available laptop computers, with caregiver and youth completing their respective individual separate sessions; the small group arm consisted of 10–12 families (separate sessions for caregivers and youth) completing their in-person sessions in small group format, led by program implementers facilitating the sessions; families assigned to the literature control arm received, via home mailings, brochures, with separate envelops designated for parents/caregivers and youth, that covered similar topics included in the other two conditions. The trial aimed to assess whether the e-Health program platform was effective and comparable to the small-group format, and if both e-Health and small-group implementation were more effective than receiving the program via mailed brochures. We also evaluated families’ engagement in and adherence to participating in a computer-driven preventive intervention program.

Results indicated that, independent of caregiver/parent age, education, or SES, families in the technology arm showed higher program initiation, lower attrition, and greater session attendance than the traditional group [169]. These findings suggest that PAAS e-Health offers an additional platform to increase families access to preventive intervention programs. PAAS-induced positive parenting practices improved youths’ self-regulation, risk-resistant decision-making, and emotional regulation during late middle childhood and pubertal transition. These gains, in turn, reduced behaviors associated with HIV/AIDS and substance use risk. Additional benefits included coping skills to navigate racial discrimination that in turn reduced depression and anxiety, as well as fostered improvements in academic performance.

We also examined the effectiveness of PAAS e-Health in inducing neural changes in Black youth and their caregivers recruited across Southern California. The study focused on PAAS e-Health effects on neural responses to reward opportunities [154] and the mechanisms underlying intervention-induced changes in protective factors, such as racial pride, that promote behavioral regulation. This study represents the first integration of prevention science and neuroscience to document intervention-induced changes in brain functionality (e.g., fMRI measures of cognitive and emotional regulation) related to intrapersonal protective processes [170].

**Fig. 2:**
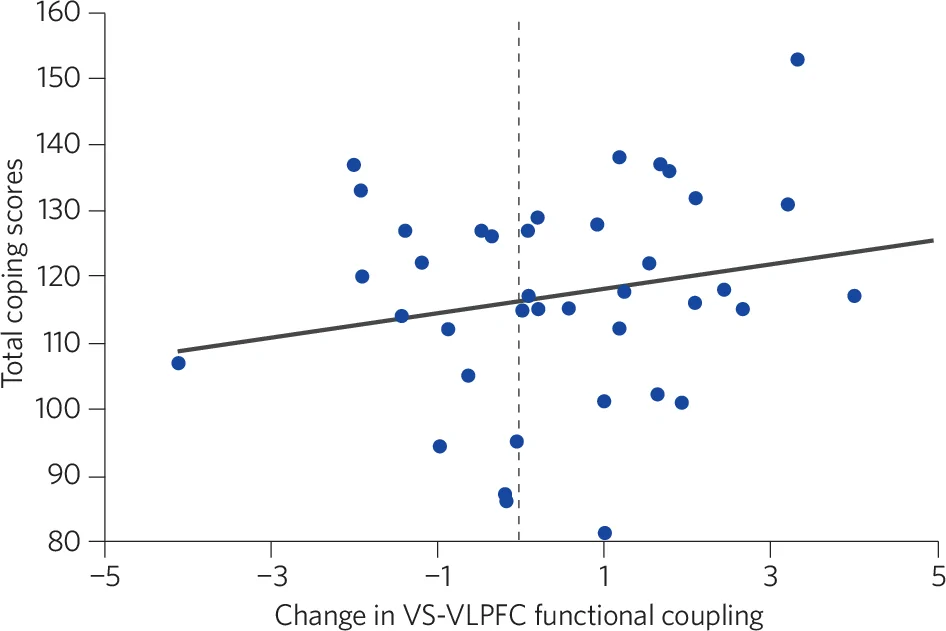
Pre-Posttest effects of PAAS e-Health on VS and VLPFC.

**Fig. 3:**
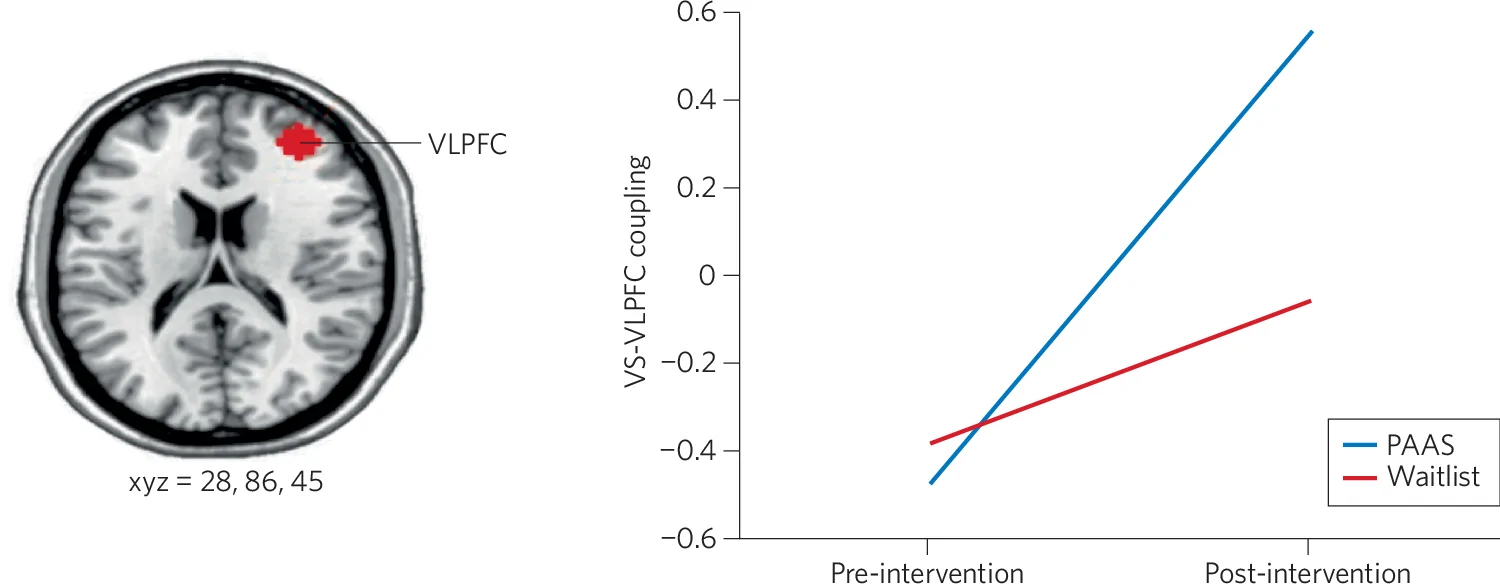
PAAS e-Health effects on Functional Coupling.

**Fig. 4:**
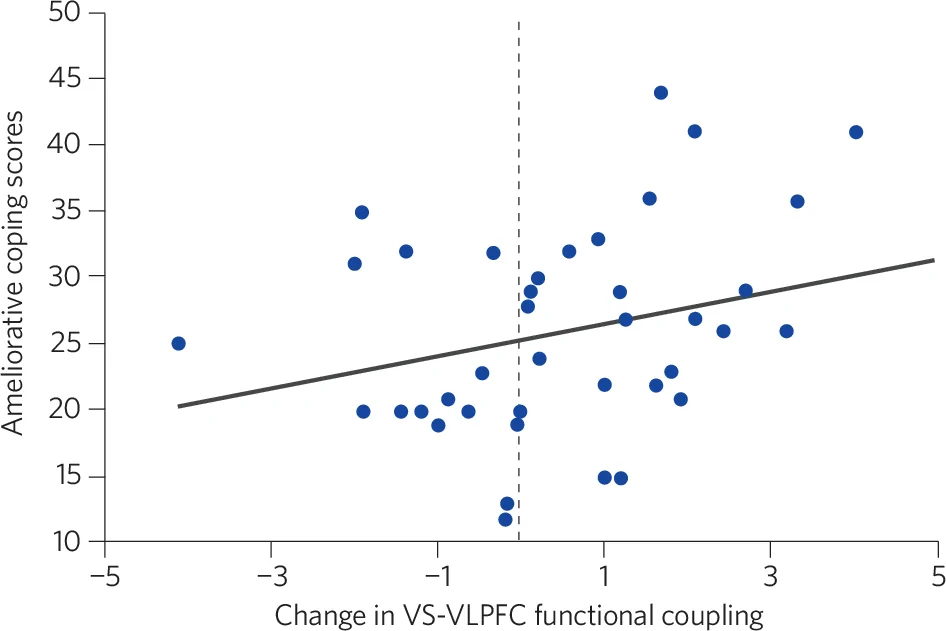
PAAS e-Health effects on Adaptive Stress Coping Processes.

Youth underwent a reward-seeking task before and after the intervention. Results showed that PAAS participants [t(21) = 2.88, *p* ≤ 0.01], but not waitlist youth [t(24) = −0.69, NS], exhibited significant increases in VS-ventrolateral PFC (VLPFC) functional coupling (Fig. 2). Increased coupling correlated with improved coping at follow-up [*B* = 2.20, SE = 1.06, *p* ≤ 0.05] (Fig. 3) and with adaptive coping strategies such as problem-solving [*B* = 1.14, SE = 0.51, *p* ≤  0.05] (Fig. 4). No significant associations were observed within individual groups.

PAAS e-Health, a culturally strength-based, family-focused life-skills program, may enhance brain functionality to support coping and mitigate the effects of structural racism. Strengthened VS-VLPFC coupling may facilitate proactive emotional, cognitive, and self-regulation in Black youth. Functional connectivity changes mediated improvements in self-regulation targeted by PAAS, reducing maladaptive, risk-engaging behaviors.

These findings provide a neurobiological framework for preventive interventions addressing risk behaviors in vulnerable populations. Tailoring interventions to individual neural profiles may optimize outcomes, and adjunctive pharmacological, psychosocial, or neurofeedback strategies could target specific circuits to further reduce risk behaviors. Furthermore, integrating behavioral interventions into precision health frameworks may enable the development of biopsychosocial profiles to efficiently guide interventions and induce neurobiological change.

## Future Directions and Clinical Implications

This review synthesizes research on the effects of structural racism on adolescents of African descent, emphasizing its implications for development, behavioral, mental, and physical health outcomes, as well as brain functionality. The key findings of this review highlight four critical themes: (1) the neurobiological and psychological consequences of prolonged exposure to racial stressors, (2) the role of social and historical contextual influences by subpopulation, (3) the mitigative effects of protective factors on brain structure and behavioral/mental health; and (4) the application of preventive interventions to prepare families and youth to navigate the consequences of structural racism through strength-based cultural assets. These findings contribute to a growing body of literature that underscores the urgent need for policies and preventive interventions that address the deeply embedded nature of structural racism and its impact on adolescent development.

A summary of themes emerging in our review is presented in Table 1. One cross-cutting theme is that structural racism has spillover effects shaping adolescents’ social environments, including their self-perceptions, stress responses, and overall well-being. The compounded effects of othering, discrimination, marginalization, and systemic inequities influence both neurobiological and psychological processes, contributing to vulnerabilities and disparities in behavioral, mental, and physical health.

**Table 1:** Structural Racism Effects and Consequences Trajectories Across Multiple Systemic Domains: Implications for Black Youths’ Development.

Domain of Influence	Structural Racism Mechanisms	Psychosocial/Neurodevelopmental Outcomes	Protective/Resilience Factors	
Neighborhoods & Built Environments	Redlining, environmental toxins, limited green space	Cognitive impairment, mental health disorders, reduced brain volume	Urban greening, housing reform	
Education Systems	Segregation, teacher bias, underfunding	School disengagement, lower academic self-efficacy	Racial pride socialization, inclusive school practices	
Healthcare Access & Delivery	Historical mistrust, racialized treatment, access barriers	Health disparities, underdiagnosis, physiological stress	Culturally competent care, community health interventions, community-engaged coalitions building	
Policing & Criminal Justice	Over-policing, CPS overreach, early exposure	Increased allostatic load, trauma responses, accelerated aging	Advocacy, juvenile diversion, legal reform, coalition building among health care professionals, community influencers, and police departments	
Social Media & Virtual Spaces	Online racial discrimination, cultural alienation	PTSD, anxiety, identity fragmentation	Digital wellness programming, ethnic peer networks, policies to reduce algorithm biases	
Biological Embedding	HPA axis dysregulation, neuroinflammation, epigenetic changes	Altered connectivity, accelerated brain aging	Cultural assets, strength-based family-youth centered preventive interventions to enhance protective coping strategies	
Family & Cultural Socialization	Anti-Black narratives, acculturation pressure	Identity confusion, internalized racism		Ethnic-racial socialization, tri-cultural identity support

The neurobiological and psychological consequences of chronic exposure to structural racism are complex. Repeated encounters with discrimination contribute to toxic stress, which disrupts neural pathways associated with emotional regulation, stress response, and executive functioning. Prolonged activation of the stress response system can lead to heightened anxiety, depression, and difficulties in cognitive processing, ultimately affecting academic performance and social interactions [140, 143]. Over time, these neurobiological changes increase vulnerability to mental health disorders and other adverse health outcomes, underscoring the need for early intervention and support. However, the consequences of structural racism effects across populations of Black youth are not homogenous, as context, exposure, and perceptions may differ as a function of immigration status [13, 14]. Therefore, unique factors and processes that contribute to variability in the effects of structural racism on mental, behavioral, and physical health outcomes of Black youth must be examined by subpopulation, accounting for the intersectional identities based on culture, racial/ethnic identity, historical, geographical, and social contexts. Further, understanding the mechanisms through which structural racism filters through to affect proximal systems of Black youth, that in turn influence when, how, and where structural racism is experienced requires a more in-depth examination of not only the nuances of internalized racialized experiences but also greater consideration of different and shared challenges within and between native-born and foreign-born Black youth.

We present a model to guide future inquiries to address these gaps (See Fig. 1). While both groups navigate systemic inequities, their historical, cultural, and migratory contexts may shape pathways that converge and others that diverge. Moreover, several factors, such as the meaning of racism and related experiences, family socialization practices regarding racism and acculturation (e.g., racial socialization and cultural socialization; and sociopolitical socialization), as well as identity formation and sense of racial-ethnic group belongingness, may have differential purposes and in turn effects on mental/behavioral health outcomes.

Black youth in the U.S. experience racism through different historical, cultural, and social lenses. These factors influence their coping mechanisms and health outcomes. Native-born Black youth often contend with the intergenerational trauma of enslavement, vestiges of Jim Crow segregation, and systemic oppression, leading to specific parenting protective strategies, such as racial socialization and collective resistance. In contrast, foreign-born, immigrant Black youth navigate challenges related to vestiges of colonialism, imperialism, economic deprivation, migration, acculturation, and xenophobia that shape distinct racialized experiences and factors and processes that inform and influence racial-ethnic identity development. These experiences also impact how both families and youth engage in socialization practices to prepare youth to grow up in a racially oppressed society. Native-born Black youths’ parents often emphasize racial pride while preparing their children for discrimination, a practice, racial socialization, rooted in collective historical resilience [54]. Meanwhile, Black immigrant families may adopt varied approaches based on their cultural backgrounds and parental encounters with racism, sometimes emphasizing ethnic identity over racial identity or downplaying racial discrimination to promote assimilation [58]. As a result, identity formation may unfold differently between the two groups. Black foreign-born youth often grapple with the duality of “model minority” and being grouped with negative socio-political, stereotypical images of their Black native-born peers. In addition, their sense of belonging as a Black American may be met with experiencing differentiation from their Black native-born peers, often driven by based on language or country of origin [56–59]. Thus, racial-ethnic identity development is further complicated by acculturation stress and the challenge of balancing the cultural expectations of the country of origin with those of the country of destination.

Protective factors may mitigate some of the negative effects of structural racism on youth well-being [166, 167]. Both groups of Black youth, native-born and foreign-born, face common exposure to structural racism across multiple domains, including schools, criminal systems, healthcare settings, and social media. Such experiences increase the risk for mental health concerns, including anxiety and depression. Both groups also encounter significant barriers to mental health support, particularly due to the lack of culturally responsive services and racially diverse clinicians to address their unique experiences. However, resilience strategies can provide critical buffers against these stressors [54]. Community networks, faith-based organizations, and peer support systems often serve as protective settings, though their effectiveness may vary based on cultural context and accessibility [171].

Family-based preventive interventions, such as The Strong Families, The Pathways for African Americans Success, and Healing Ethno-Racial Trauma, Culturally Informed Parent Training are programs that have demonstrated effectiveness in enhancing strength-based cultural assets that assist youth in navigating the potential harm effects of structural racism [109, 110, 168, 169]. For example, strong racial-ethnic identity development fosters self-esteem and resilience, helping Black youth navigate racialized experiences with a sense of purpose and internal control, thereby reducing both mental and physical health vulnerabilities. Community support, including mentorship, peer networks, and faith-based organizations, can also provide essential social buffers against stress. Additionally, culturally responsive clinical interventions—such as therapy that acknowledges racial trauma and school policies that promote inclusivity—can help counteract the effects of systemic discrimination. Addressing these protective factors is crucial for fostering positive developmental outcomes and may also reduce disparities that in turn advance health equity for Black youth.

## Implications for Research

Several indistinguishable patterns emerged in our review. Limited attention given to within and between group heterogeneity, including gendered effects, suggesting need for sample representativeness to more accurately capture the consequences and nuances of structural racism effects on youth of African descent. Future research studies explored how neuroscientific and biopsychosocial mechanisms emerging from structural racism influence adolescents’ interactions and experiences in multiple settings, including schools, medical care settings, and virtual social media platforms. In addition, there was an omission of studies that captured the unique nuances of how structural racism gets under the skull of adolescents of both groups, and its prognostic significance for long-term academic, behavioral, and health disparities. Investigating these pathways will provide deeper insights into how systemic inequities shape cognitive, emotional, social, and behavioral outcomes, ultimately informing more targeted interventions and policies to support the well-being of all Black youth.

There is also insufficient longitudinal data to track the long-term impact of structural racism on adolescent overall social emotional and brain development to predict health outcomes over the life course by subpopulation. There is a need for greater funding investment in longitudinal studies research to address this methodological gap. In addition, factors such as gender, socioeconomic status, and immigrant status intersecting with racial discrimination have a compounded effect on mental health outcomes that are understudied and warrant further investigation. Comparative studies are also needed to disentangle the experiences of structural racism between Black native-born and Black foreign-born, immigrant youth, including the significance of geographic context, such as rurality versus urban versus suburban. Findings from these studies may hold promise to inform both upstream and downstream preventive interventions. Finally, the voices and lived experiences of these youth and their families should be centered in these longitudinal studies, through participatory and qualitative methodologies.

## Implications for Practice

Effective practice considerations must prioritize trauma-informed and culturally responsive approaches when working with Black youth, both native-born and foreign-born. Structural racism is deeply embedded in every aspect of our society---education, healthcare, social media, law enforcement, and economic systems, shaping the developmental trajectories, brain development, behavioral, and mental health of adolescents of African descent. In educational settings, disparities in school discipline, tracking into lower academic levels, and limited access to advanced coursework create barriers to success, reinforcing systemic inequities, elevating risks for early involvement in criminal systems. Schools and community organizations can play a critical role in fostering resilience by implementing anti-racist curricula and providing support services that acknowledge and address the impact of racial trauma on youth development and mental health. Creating inclusive educational environments can help mitigate the adverse effects of discrimination while promoting positive identity development [172]. Similarly, as healthcare disparities, including the underdiagnosis of mental health conditions and limited access to culturally competent care, contribute to poorer health outcomes [173], health care providers, including clinical practitioners should integrate trauma-informed care as a standard framework to address the psychological and emotional impact of racial stress. Additionally, community organizations as well as parental and family support programs are essential in equipping caregivers with the tools to navigate conversations about racism and racial identity with their children [76, 174].

These programs can strengthen families’ skills and capacities to foster open dialogue, adaptive coping strategies, and build protective factors that support adolescent well-being. Ideally, upstream changes would eliminate the need for families and Black youth to be resilient. Given the current climate and low prospects for the elimination of structural racism, investing in strength-based preventive interventions will be critical to prepare families for the society in which their children will grow up in. Encounters with law enforcement, from school policing to racial profiling, further expose Black youth to stress and trauma, shaping their perceptions of safety, social justice, and equity. Implementing evidence-based community policing strategies, a practice occurring in several urban municipalities may improve both perceptions of public safety and police-community relationships [175]. Efforts to address economic disparities are also needed, including eliminating policies and practices that limit access to wealth-building opportunities and stable employment for families. These barriers contribute to and exacerbate stressors that influence adolescent development.

Economic development programming, such as job training for youth, are effective avenues to promote life skills that prepare youth for job attainment [176]. Together, these practical strategies may hold promise to create upstream intervention with downstream consequences that are supportive and equitable for Black youths’ optimal development.

## Implications for Policy

To address the experiences of structural racism among Black youth, several policy recommendations are essential. First, ensuring equitable access to mental health resources requires increased funding for community-based services specifically tailored to the needs of Black youth. Culturally responsive and accessible mental health care can help mitigate the psychological and neurobiological effects of chronic racial stress. Additionally, educational equity initiatives must be implemented to reduce disparities in school discipline, academic tracking, and access to advanced coursework. Addressing these systemic inequities will create more supportive learning environments that foster academic success and well-being. Finally, broader systemic reforms are necessary to dismantle the structural barriers that perpetuate racism and lead to racial inequities in health, education, and economic mobility. Comprehensive policy changes should target the root causes of these disparities, promoting long-term, upstream, sustainable progress toward racial justice and equity [177].

## Conclusion

This review underscores the pervasive effects of structural racism on Black native-born and Black foreign-born youths’ adjustment, behavioral, mental, and physical health. There is a critical need for systemic change to reduce the mental health burden on Black youth. Advancing health equity requires a multifaceted approach that centers youth voices, including continued research, culturally informed interventions, and systemic policy changes to promote health equity and reduce mental health disparities in Black youth populations.
